# Patients with Acute Limb Ischemia Might Benefit from Endovascular Therapy—A 17-Year Retrospective Single-Center Series of 985 Patients

**DOI:** 10.3390/jcm12175462

**Published:** 2023-08-23

**Authors:** Kerstin Stoklasa, Sabine Sieber, Shamsun Naher, Bianca Bohmann, Andreas Kuehnl, Thomas Stadlbauer, Heiko Wendorff, Gabor Biro, Michael A. Kallmayer, Christoph Knappich, Albert Busch, Hans-Henning Eckstein

**Affiliations:** 1 Department for Vascular and Endovascular Surgery and Munich Aortic Center (MAC), University Hospital Rechts der Isar, Technical University Munich (TUM), Ismaninger Str. 22, 81675 Munich, Germany; kerstin.stoklasa@usz.ch (K.S.); sabine.dallmann-sieber@mri.tum.de (S.S.); ge86poh@mytum.de (S.N.); bianca-bohmann@web.de (B.B.); a.kuehnl@tum.de (A.K.); thomas.stadlbauer@usz.ch (T.S.); heiko.wendorff@mri.tum.de (H.W.); gabor.biro@mri.tum.de (G.B.); michael.kallmayer@mri.tum.de (M.A.K.); christoph.knappich@mri.tum.de (C.K.); albert.busch@uniklinikum-dresden.de (A.B.); 2 Department of Vascular Surgery, University Hospital Zürich, Rämistrasse 100, 8091 Zürich, Switzerland; 3 Division of Vascular and Endovascular Surgery, Department of Visceral, Thoracic and Vascular Surgery, Medical Faculty Carl Gustav Carus and University Hospital, Technical University Dresden, 01307 Dresden, Germany

**Keywords:** acute lower limb ischemia (ALI), mortality, complications, risk factor, endovascular revascularization, embolectomy/thrombectomy, bypass surgery

## Abstract

Acute lower limb ischemia (ALI) is a common vascular emergency, requiring urgent revascularization by open or endovascular means. The aim of this retrospective study was to evaluate patient demographics, treatment and periprocedural variables affecting the outcome in ALI patients in a consecutive cohort in a tertiary referral center. Primary outcome events (POE) were 30-day (safety) and 180-day (efficacy) combined mortality and major amputation rates, respectively. Secondary outcomes were perioperative medical and surgical leg-related complications and the 5-year combined mortality and major amputation rate. Statistical analysis used descriptive and uni- and multivariable Cox regression analysis. In 985 patients (71 ± 9 years, 56% men) from 2004 to 2020, the 30-day and 180-day combined mortality and major amputation rates were 15% and 27%. Upon multivariable analysis, older age (30 d: aHR 1.17; 180 d: 1.27) and advanced Rutherford ischemia stage significantly worsened the safety and efficacy POE (30 d: TASC IIa aHR 3.29, TASC IIb aHR 3.93, TASC III aHR 7.79; 180 d: TASC IIa aHR 1.97, TASC IIb aHR 2.43, TASC III aHR 4.2), while endovascular treatment was associated with significant improved POE after 30 days (aHR 0.35) and 180 days (aHR 0.39), respectively. Looking at five consecutive patient quintiles, a significant increase in endovascular procedures especially in the last quintile could be observed (17.5% to 39.5%, *p* < 0.001). Simultaneously, the re-occlusion rate as well as the number of patients with any previous revascularization increased. In conclusion, despite a slightly increasing early re-occlusion rate, endovascular treatment might, if possible, be favorable in ALI treatment.

## 1. Introduction

Acute lower limb ischemia (ALI) is a common vascular emergency with an incidence of 9–27/100.000 persons/year in the Western world [1,2,3]. It is defined by sudden onset of insufficient arterial perfusion and thus lack of oxygen with an imminent threat to the limb causing pain, sensory deficit, palsy and ultimately irreversible tissue damage [4,5].

Previous data suggest that ALI is associated with an in-hospital mortality of up to 20% and short-term limb loss rates of 11–42% [4,6,7,8] depending on the severity of disease and the time to revascularization. Due to the heterogeneity of the disease, the plethora of treatment options and the limited availability of RCTs and modern real-world data, the recent European Society of Vascular Surgery (ESVS) ALI guidelines recommend strongly that ALI should be treated in vascular centers that offer the full range of open and endovascular interventions [9].

Traditionally, open surgical procedures such as embolectomy and bypass surgery have been the treatment of choice; however, more and more endovascular procedures including aspiration/rotation thrombectomy, catheter-directed thrombolysis and transluminal angioplasty with or without stenting have been implemented in many institutions over the last couple of years.

With respect to head-to-head studies, only five randomized controlled trials comparing thrombolysis vs. open surgery and/or placebo [10,11,12,13,14] have been performed more than 20 years ago. Furthermore, only some large retrospective studies with at least 100 patients and valid data on survival and amputation rates exist [8,15,16,17,18]. Recently, Grip et al. [19] were able to show a significant benefit of endovascular therapy compared with open surgical therapy in ALI, but without considering possible influencing co-variables. However, a study from Taiwan in 2021 by Yang et al. failed to confirm a difference in outcome between thrombolysis and surgical treatment [20]. Three systemic reviews, which also focused on the treatment of ALI, also demonstrated no differences between the various treatment options [21,22,23]. It should be noted that the RCTs used were largely the same.

The aim of this study was to evaluate patient demographics, treatment modalities and periprocedural variables in patients with ALI in a large consecutive cohort over 17 years in a high-volume tertiary referral center. We hypothesize that early and midterm outcomes have changed over time due to an evolving clinical environment with an increasing use of endovascular technologies alone or in combination (hybrid procedures).

## 2. Patients and Methods

A retrospective single-center analysis of all consecutively treated patients with acute limb ischemia (ALI) at the Department for Vascular and Endovascular Surgery and Munich Aortic Center (MAC), University Hospital rechts der Isar, Technical University Munich (TUM), between January 2004 and December 2020 was performed.

### 2.1. Inclusion and Exclusion Criteria

**Figure 1:**
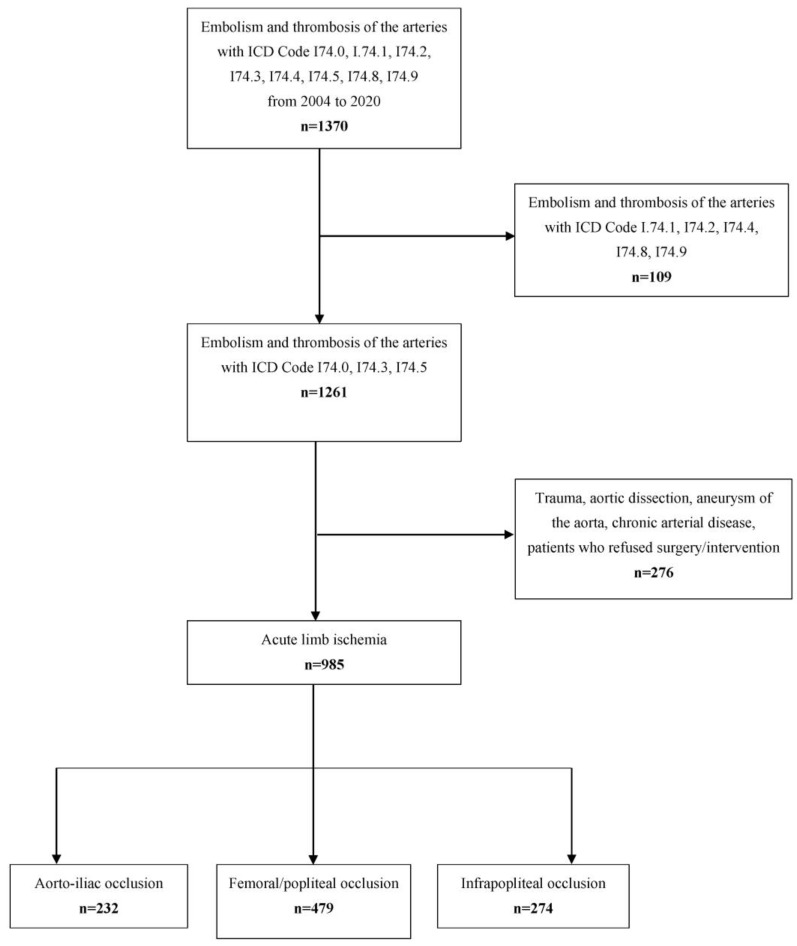
Patient flow. The total dataset contained all hospitalizations in the Department for Vascular and Endovascular Surgery and Munich Aortic Center (MAC), University Hospital rechts der Isar, Technical University Munich (TUM) in the years from 2004–2020. ICD: International Classification of Diseases (version10). One hundred and nine patients with ICD code I74.1, I74.2, I74.4, I74.8 and I74.9 (embolism and thrombosis of the mesenteric and renal arteries, arteries of the upper extremity and other undefined localization) and 276 patients with ischemia caused by trauma, aortic dissection, chronic arterial disease or without surgical treatment were excluded.

All patients with an admission diagnosis of I74.0 ((ICD-10-GM (International Classification of Disease, 10. Revision, German Modification, occlusion of the aorta and iliac arteries), I74.3 (occlusion of the femoral and popliteal artery) and I74.5 (occlusion below the knee) were identified. All patients treated by open surgery, endovascular or hybrid procedures as well as primary amputation were included. Patients with ALI due to trauma or aortic dissection and patients who refused any active revascularizing treatment were excluded from the study (Figure 1). Also excluded were patients with critical limb ischemia (peripheral artery disease [PAD] stage III and IV) without an acute component. A pre-existing PAD in the diagnosis list in combination with acute symptoms of an ischemia (acute on chronic) was not an exclusion criterion.

### 2.2. Data Acquisition

Data were obtained retrospectively from electronic patient records. For this purpose, inpatient physicians’ letters, surgical reports and also postoperative outpatient physicians’ letters as well as information from other clinics and hospitals were used to generate a comprehensive dataset. Patient demographics and comorbidities (age, sex, arterial hypertension, smoking status, peripheral arterial disease [PAD], coronary artery disease [CAD], atrial fibrillation [AF], hyperlipidemia/therapy with statins, diabetes mellitus, chronic obstructive pulmonary disease [COPD], renal insufficiency, previous cancer and obesity) were retrieved.

Regarding the definition of a pre-existing PAD, all patients coded with PAD in the electronic patient file were defined as having concomitant disease PAD. This was independent of the affected leg, as we consider PAD a multi-vessel disease. Therefore, patients with PAD Fontaine stage I and IIa, not previously treated surgically or endovascularly, were also included, as well as patients with PAD stage IIb, III and IV. Every patient with ALI received preoperative ultrasound by Doppler and duplex sonography and/or CT- or /MR-angiography to assess the localization of the occlusion and the amount of atherosclerosis.

Symptom onset and creatine kinase (CK; U/L) were assessed at admission. Additionally, any kind of previous revascularization (open surgery, endovascular treatment or hybrid procedures) of the index limb was identified. The ASA-Score was used to assess the patients’ physical condition (ASA—American Society of Anesthesiologists). A pre-diagnosed hyperlipidemia with established statin therapy was consolidated as hyperlipidemia.

### 2.3. Classification of Acute Limb Ischemia and Treatment Modalities

The extent of ALI was classified according to the Trans-Atlantic Inter-Society Consensus (TASC) classification, which is based on the Rutherford protocol [24] and was determined based on the presenting symptoms at inpatient admission. Symptom duration (h, hours) was defined as the time interval between symptom onset and the start of the revascularizing treatment [24]. Occlusion levels were determined by duplex ultrasound (DUS), MR-angiography (MRA), CT-angiography (CTA) and possible intraoperative findings and were classified as aorto-iliac, femoral, popliteal or infrapopliteal level of occlusion. Etiology of ALI was clinically distinguished between arterial thrombosis, embolic occlusion or an occluded or freshly embolizing or occluding popliteal artery aneurysm (PAA). If PAD was present in the past [PAD stage I–IV], an acute on chronic thrombosis was assumed. In patients with acute onset of pain without collateral circulation present in the preoperative imaging and known or newly diagnosed AF, sepsis or endocarditis, arterial embolism was assumed as the most likely cause.

Treatment modalities were applied according to the clinical condition of the patient, the anatomic extent of the disease, the availability of endovascular tools and the vascular surgeon’s treatment preference. We differentiated between open surgical procedures (embolectomy, thrombectomy, bypass surgery by vein or graft interposition, endarterectomy), purely endovascular procedures (thrombolysis, aspiration/rotating thrombectomy, balloon angioplasty or stenting), hybrid procedures (open surgery plus any endovascular treatment) and primary amputation (no simultaneous revascularization attempt or revascularization of the deep femoral artery only to ensure wound healing of the amputation wound).

In all patients who received lysis, angiography was performed after 24 h. If angiography was satisfactory after sometimes additionally performed PTA/stent, lysis was terminated. If thrombotic material was still detected, lysis was continued for another 24 h and angiography was performed again the following day as a standard of care.

Additionally, we assessed the need for lower leg fasciotomy (categorized into immediate (intraoperative) and delayed).

Revascularization was considered successful if at least one patent outflow vessel at the lower leg was achieved as assessed by intraoperative control angiography. When angiography was not possible (cardiopulmonary instability, extensive surgical care, long operative time, renal insufficiency) clinical assessment of the revascularized leg (appearance/color, recapillarization time, clinical improvement of sensory and motor deficit), as well as the quality of the acquired Doppler signals were included. In addition, an ankle-brachial index was performed as postoperative routine. Any amputation above the heel was considered as “major amputation”.

### 2.4. Outcome Events

The *primary outcome events* (POE) were the combined 30-day mortality and major amputation rate of the index leg (safety endpoint) and the combined 180-day death and major amputation rate of the index leg (efficacy endpoint).

The *secondary outcome events* included systemic (medical) and surgical (leg-related) in-hospital complications and 5-year mortality and major amputation rate of the index leg. Additionally, the early (in-hospital) re-occlusion rates and their consecutive management was assessed. In order to be able to give a trend for the long-term course, factors influencing a worse or better outcome were also examined.

Systemic complications were defined as follows: renal: any worsening of the preoperative stage of renal function based on KDIGO (Kidney Disease Improving Global Outcomes), cardiac: myocardial infarction/heart failure, pulmonary: pneumonia/respiratory failure. Furthermore, we assessed the rates of rhabdomyolysis. Major adverse cardiac event [MACE] was defined as myocardial infarction and/or death [25].

Surgical in-hospital leg-related complications were: any surgical site infection (SSI), seroma/lymphatic fistula, re-occlusion, new symptoms of acute limb ischemia or bleeding requiring surgical treatment.

To look for temporal trends, the patient population was divided into five equal-sized blocks (first to fourth 200 and last 185), corresponding roughly to the years 2004–2007, 2008–2010, 2011–2013, 2014–2016, and 2017–2020, respectively. Major adverse limb event [MALE] was defined as loss of patency of the revascularization, re-intervention on the revascularized segment or major amputation of the revascularized limb [25].

### 2.5. Statistics

For the descriptive analysis, median and first and third quartile (Q 1–3) were reported for variables with skewed distribution. For categorical variables, frequency and percentage were given. For each patient, information on the last follow-up date existed, from which the outcome could be calculated (endpoint reached yes/no). Trends for categorical variables were tested by the Chi-squared test for absolute numbers. For continuous variables, the Pearson correlation coefficient was used. To analyze different influences on the POE, a stepwise univariate and multivariable Cox regression model using Wald Test was used. Results are given as forest plots with adjusted Hazard Ratios (aHR—adjusted for all factors within the model).

Kaplan–Meier curves were generated for amputation-free survival as a function of the appropriate treatment given at 30 days, 180 days and 5 years. In order to compare the influence of the different treatment methods, a pairwise comparison using Log-Rank test was also performed.

Statistical analyses were performed using R version 4.0.3 on Windows, and forest plots were created using metafor package. All *p*-values were two-sided with an alpha-level of 5%.

## 3. Results

### 3.1. Patient Cohort and Initial Presentation

A total of 1370 patients with the diagnosis “Embolism and thrombosis of the arteries” with ICD Code I74.0-I74.9 were treated between 1 January 2004 and 31 December 2020. After excluding 109 patients with ICD code I74.1, I74.2, I74.4, I74.8 and I74.9 (embolism and thrombosis of the mesenteric and renal arteries, arteries of the upper extremity and other undefined localization) and 276 patients with ischemia caused by trauma, aortic dissection, or chronic arterial disease or without surgical treatment, 985 patients remained eligible to be included in this analysis (Figure 1). Thirty-day follow-up was completed by 924 patients; 180-day follow-up was completed by 734 patients. The median age of this cohort was 71 years (Q 1-3 62–80) (56.3% male). The median onset of symptoms was 24 h (IQR 6–72). According to the TASC classification, 27%, 35%, 34% and 4% of patients were graded as TASC I, IIa, IIb and III, respectively (Table 1) and this was determined at the time of inpatient admission.

Most patients had arterial thrombosis (64%) or embolic occlusion (31%), while PAA-related ALI was observed in 5% of the patients. Detailed patients’ characteristics are given in Table 1. Approximately half of the cohort (51%) had previous revascularization procedures at the index leg. The femoro-popliteal segment was the most frequent diagnosed occlusion site.

While the patient age did not change during the observation period, symptom onset significantly prolonged and PAD as pre-diagnosis increased (Supplementary Table S1). On the other hand, patients presented with a higher percentage of lower grade of ischemia and a significantly higher percentage of patients had any previous revascularization (32%, 1st quintile to 70.8%, 5th quintile, *p* < 0.001, Table 2).

Accordingly, arterial thrombosis was the most common etiology of ALI in the later years (Table 2). The proportion of infra-popliteal pathologies grew over time, although femoral and popliteal occlusions remained the most common site of occlusion.

Median length of hospital stay was 10 days (Q 1-3 6–18, Table 3). However, the length of hospital stay decreased over time (1st quintile: 11 to 5th quintile: 10 days, *p* = 0.020, Supplementary Table S1).

### 3.2. Procedural Management

All patients were treated emergently. Open surgical revascularization (54%) was most frequently performed followed by hybrid (24%) and endovascular-only (20%) procedures. Primary amputation was necessary in 20 patients (2%). Fasciotomy was performed in 36% of the cohort (83% immediate). The median procedure time was 150.5 min (IQR 90–260.75, Table 3).

**Figure 2:**
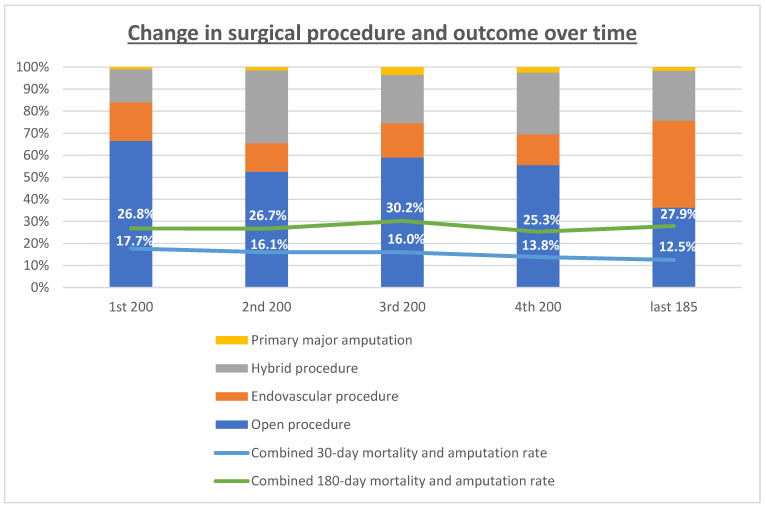
Treatment modalities and primary outcome events at 30 days (safety endpoint) and at 180 days (efficacy endpoint) over time. The percentages of the respective treatment modality and major amputation rate in the quintiles of patients is presented. The trend curves for POEs are displayed.

Treatment modalities changed over time with an increase in endovascular therapy from 17.5% in the 1st quintile to 39.5% in the 5th quintile (*p* < 0.001) and a parallel decrease in open surgical procedures from 66.5% to 36.2%, respectively (*p* < 0.001). The percentage of hybrid procedures remained relatively stable with 15% and 22.5%, respectively (Table 2, Figure 2).

Patients treated with catheter-directed thrombolysis (n = 102) were analyzed separately. Here, 84 patients were treated for 24 h by thrombolysis followed by angioplasty until a complete revascularization could be achieved (82%). In 18% of cases, prolonged thrombolysis for 48 h followed by endovascular optimization was necessary until complete revascularization was achieved (Table 3).

After the first procedure, 184 (19%) of all patients suffered from partly asymptomatic or just minor symptomatic re-occlusion and 105 patients (11%) had new ALI symptoms during the hospital stay. Of the 289 patients, 219 required re-operation because of high level of suffering or symptoms of new acute ALI (Table 3, Supplementary Table S2). Thirty-two patients were amputated and 10 patients did not receive any further therapy, while the remaining patients underwent additional revascularization procedures. The MALE (loss of patency of the revascularization, re-intervention on the revascularized segment or major amputation of the revascularized limb) rate was therefore 30.5%.

Regarding the subsequent treatment regime after early occlusion, major amputation was most frequently required after primary open surgical therapy (21%). After initial endovascular therapy, open/hybrid conversion was done in 44%, while 36% of patients could be treated again by endovascular means (Supplementary Table S2).

### 3.3. Outcome Analysis

The combined 30-day mortality and major amputation rate was 15% (POE: safety endpoint); the combined 180-day mortality and amputation rate was 27% (POE: efficacy endpoint). Eighty patients (9%) died during the first 30 days, due to cardiac events (39.6%), multiple organ failure/sepsis (45.3%, 13.2% with crush syndrome) and pulmonary failure (7.5%). Major amputation was performed in 79 patients (9%) during the first 30 days, either primarily (2%) or due to re-occlusion and/or the initial extent of ALI (7%, Table 3). The combined mortality and amputation rates at 30 and 180 days were higher after open surgery (62.4%/60.7%) and hybrid procedures (21.3%/22.9%) compared to endovascular treatment (8.5%/10%) and primary amputation (7.8%/6.4%, Table 3).

The results of the analysis over time showed a continuous, although not significant, decrease in 30-day mortality and amputation rates (1st quintile 17.1%, 2nd quintile 16.1%, 3rd quintile 16.0%, 4th quintile 13.8%, 5th quintile 12.5%; *p* = 0.136). After 180 days, this effect could no longer be demonstrated (1st quintile 26.8%, 2nd quintile 26.7%, 3rd quintile 30.2%, 4th quintile 25.3%, 5th quintile 27.9%; *p* = 0.946, Table 2).

With respect to in-hospital complications, cardiovascular events were observed in 13% of the patients, followed by respiratory failure in 11% and renal failure in 9% (3% with permanent dialysis). Rhabdomyolysis occurred in 5% of the patients. SSI occurred in 19%, bleeding in 4% and a seroma/lymphatic fistula in 6% of the patients (Table 3). The MACE (myocardial infarction and/or death) rate was therefore 30.5%.

### 3.4. Influence of Treatment Modalities on Primary Outcome Events over Time

In parallel with the evolving treatment modalities with an increase in endovascular therapy and a parallel decrease in open surgical procedures, the combined 30-day mortality and amputation rate tended to decrease, as mentioned above, despite the significantly higher number of patients with previous ipsilateral revascularizations (1st quintile 32% to 5th quintile 70.8%, *p* < 0.001). However, the percentage of in-hospital reocclusions increased significantly in the last quintile (1st quintile: 16% to 5th quintile: 33.5%, *p* < 0.001, Table 2).

**Figure 3:**
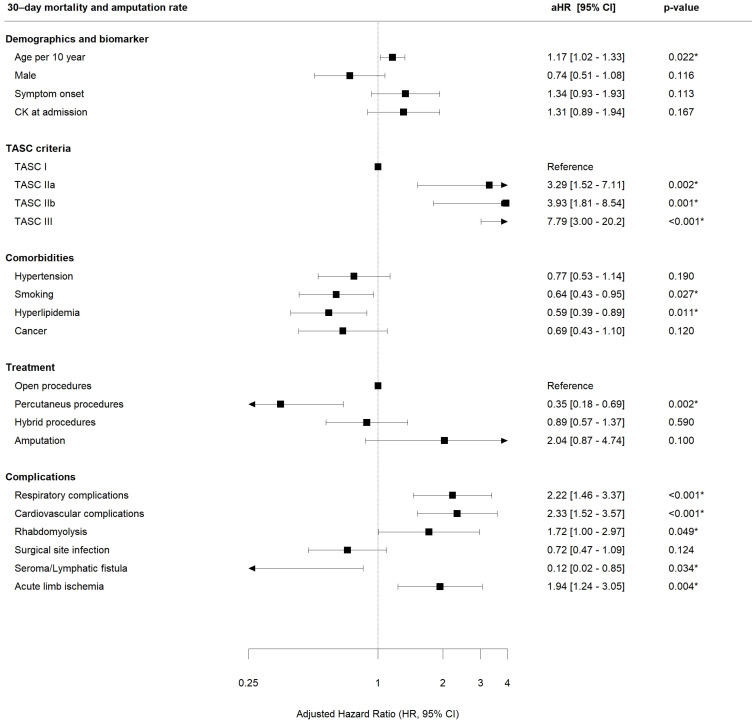
Association of acute limb ischemia (ALI) demographics, treatment modality and complications on combined 30-day mortality and major amputation rate (primary safety endpoint, multivariate regression analysis *). * HR adjusted according to the other variables within the model. Stratification based on re-occlusion. *p* < 0.05 is considered significant and highlighted with asterisk. Multivariate Cox proportional hazard model was done using Wald Test. ASA CK, Creatine kinase (standard value < 170 U/mL); TASC, Trans-Atlantic Inter-Society Consensus.

Upon multivariable analysis, the use of percutaneous endovascular procedures alone was associated with a significantly decreased combined 30-day death or major amputation rate as compared to solely open surgical procedures (aHR 0.35, CI 0.18–0.69, *p* = 0.002, relative risk reduction 65%, Figure 3).

**Figure 4:**
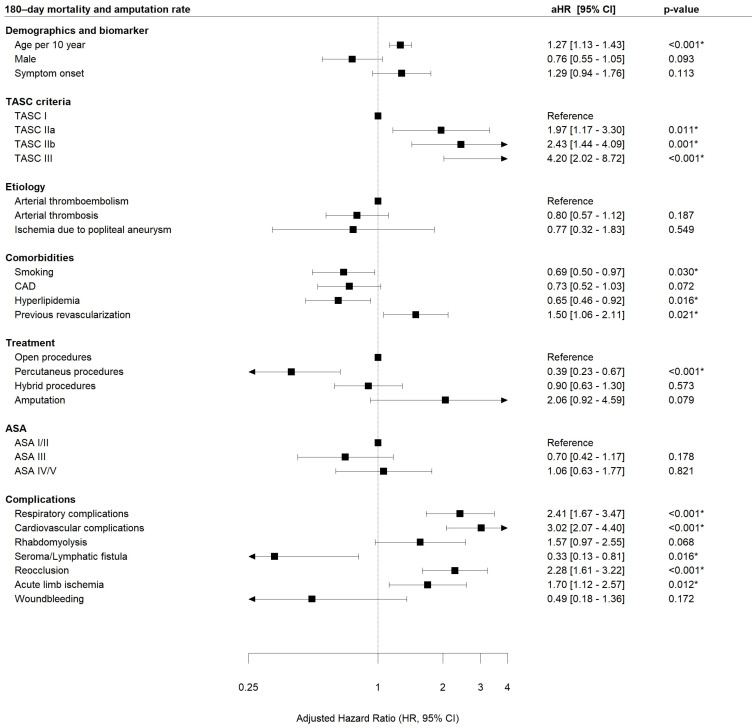
Association of acute limb ischemia (ALI) localization, treatment modality and complications on combined 180-day mortality and major amputation rate (primary efficacy endpoint, multivariate regression analysis *). * HR adjusted according to the other variables within the model. No stratification was needed. *p* < 0.05 is considered significant and highlighted with asterisk. Multivariate Cox proportional hazard model was done using Wald Test. ASA, American Society of Anesthesiologists; CAD, coronary artery disease; TASC, Trans-Atlantic Inter-Society Consensus, h = hours, complication—acute limb ischemia means new acute ischemia of the index leg.

Similar results were seen after 180 days (endovascular: aHR 0.39, CI 0.23–0.67, *p* < 0.001, relative risk reduction 61%, Figure 4) as well as after 5 years (endovascular: aHR 0.60, CI 0.40–0.91, *p* = 0.017, relative risk reduction 40%, Supplementary Figure S1).

**Figure 5:**
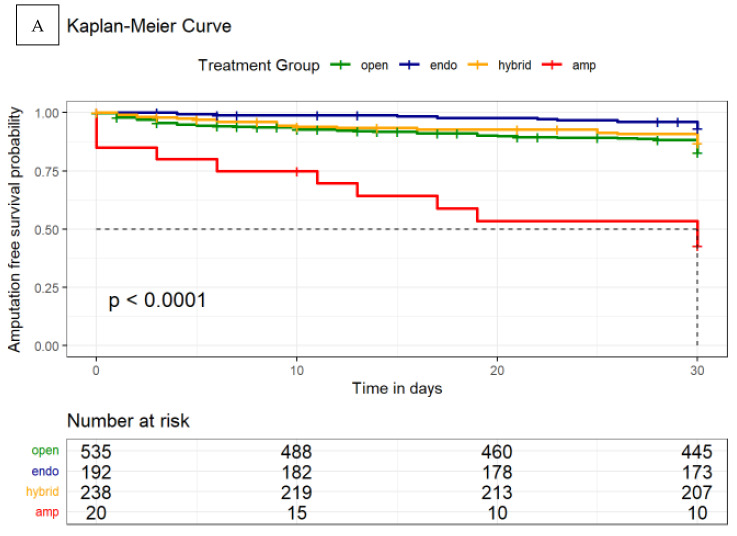

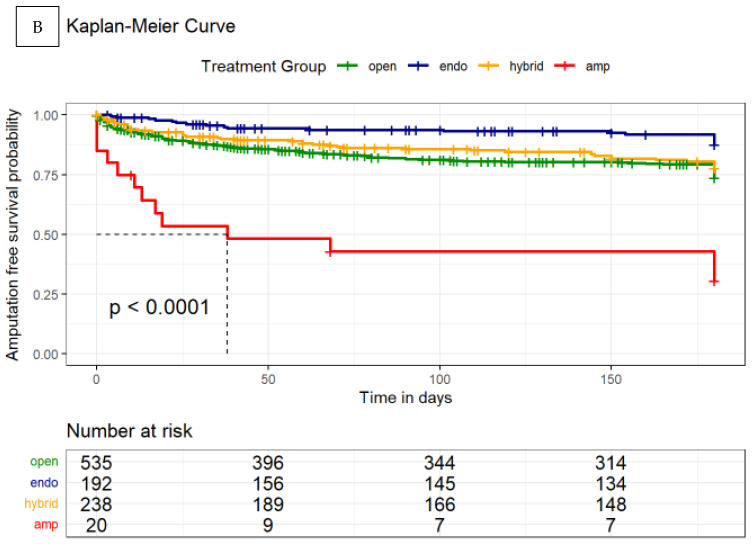
Kaplan–Meier Curves of combined mortality and/or amputation rate stratified by treatment modality. (**A**) 30-day mortality and major amputation rate (safety endpoint) and (**B**) 180-day mortality and major amputation rate (efficacy endpoint). *p* refers to the context of all treatment modalities. *p* < 0.05 is considered significant. Log-rank test was used.

Kaplan–Meier analysis also showed a significant difference in amputation-free survival between the different treatment modalities at 30 days (*p* < 0.0001, Figure 5). Additionally, a pairwise comparison using log-rank test was performed to determine more precisely which treatment method had a significant effect in Kaplan–Meier analysis. Pairwise comparison of the therapy methods confirmed a significantly better survival for endovascularly treated patients (*p* < 0.001 compared to open surgical procedures; *p* = 0.035 compared to hybrid procedures); patients who received a primary amputation had a significantly worse outcome compared to any revascularization procedure *p* < 0.001, Supplementary Table S5). Similar results were also confirmed after 180 days and 5 years (Figure 5 and Figure S2, Supplementary Table S5).

To further investigate the effect of revascularization modality on outcome, another Kaplan–Meier analysis was performed comparing endovascular vs. open vs. hybrid procedure. This analysis also showed superiority of endovascular therapy in terms of amputation-free survival at 30 and 180 days and 5 years. Unfortunately, due to the long observation period, the number of people at risk decreased considerably after 5 years (Supplementary Figures S2 and S3).

Any severe leg-related or systemic complication except SSI and seroma/lymphatic fistula was significantly associated with an increased likelihood of death of amputation during the short- and midterm interval (Figure 3 and Figure 4).

With respect to clinical variables, older age (30 d: aHR 1.17; 180 d: 5 y: aHR 1.39) and a more severe ALI stage (30 d: TASC IIa aHR 3.29; TASC IIb aHR 3.93; TASC III aHR 7.79; 180 d: TASC IIa aHR 1.97; TASC IIb aHR 2.43; TASC III aHR 4.2; 5 y: TASC IIa aHR 1.76; TASC IIb aHR 2.59; TASC III aHR 5.33; as compared to TASC I) were significantly associated with worse outcomes (Figure 3, Figure 4, and Figure S1). In contrast, hyperlipidemia and already established statin therapy was significantly associated with better outcomes at 30 days (aHR 0.59), 180 days (aHR 0.65) and 5 years (aHR 0.65, Figure 3, Figure 4, and Figure S1).

## 4. Discussion

In this retrospective 17-year, consecutive single-center analysis on 985 patients with acute limb ischemia (ALI), the combined 30-day (safety) and 180-day (efficacy) mortality and major amputation rates were 15% and 27%, respectively (Table 3). Endovascular ALI therapy was used increasingly over time and open surgical therapy less often (Table 2). Our multivariable analysis revealed that endovascular therapy was associated with better outcomes with respect to the combined 30-day death and major amputation rate of the index leg (primary safety endpoint, adjusted HR 0.35 [0.18–0.69] *p* = 0.002, relative risk reduction 65%) and the combined 180-day death and major amputation rate of the index leg (primary efficacy endpoint, aHR 0.39 [0.23–0.67] *p* < 0.001, relative risk reduction 61%, Figure 3 and Figure 4). Similar observations favoring endovascular treatment of acute aortic occlusions were previously demonstrated by our group [26].

Grip et al. [19] and others demonstrated a beneficial effect of endovascular treatment regarding amputation-free survival, also in the long-term follow up; however, the 1-year combined mortality/amputation remains approximately 50% in contemporary series [27]. In our analysis, considerable improvement in mortality and amputation rates after 180 days was shown, yet amputation-free survival after 180 days was 73% (Table 3).

Additionally, accounting for an endovascular first strategy, a significant increase in endovascular procedures over the last years could be shown (Table 2, Figure 2). Accordingly, a beneficiary (nonsignificant) time trend was observed for the 30-day POE (safety endpoint, Table 2), although the patient cohort did not change with respect to comorbidities and ASA stage (Supplementary Table S1). Hence, despite solid results after multivariate analysis, the possible benefit of endovascular ALI therapy might be corroborated by the changing patient characteristics with more complex previous revascularizations, a decrease in severity of ischemia and evolving endovascular techniques and skills. This turnaround of therapeutic procedures in PAD and ALI treatment was also reported in a nationwide study from Germany [28], with an increasing percentage of endovascular treatment, especially in lower stages of the disease. Here, the days of hospitalization decreased significantly, also noted in our data (Supplementary Table S1). It should be kept in mind that with the increasing number of previous revascularizations we have demonstrated, both endovascular and open surgical, due to, for example, mechanical problems (iatrogenic stenoses in the area of the anastomoses, outflow obstruction, stent rupture or mismatch, etc.), acute limb ischemia may occur more frequently. This etiology will therefore play an increasing role in the future, along with arterial thrombosis and arterial embolism.

Yet, questions remain about the ideal anticoagulation after surgical/interventional procedures, particularly considering the use of new oral anticoagulants and dual antithrombotic therapies to positively impact long-term amputation-free survival [29,30].

In this context, our analysis remarkably showed a high number (289 patients) of in-hospital re-occlusions, which increased in recent years and was associated with the significant increase in patients with previous revascularization of the index leg (Table 2). The rate of endovascular procedures has increased, but there were also more re-occlusions during the hospital stay. This may certainly be related to endovascular-first therapy, but also to the increasing number of pre-operated patients. Accordingly, our multivariable analysis showed that endovascular treatment and re-occlusion with new acute ischemia are independent variables that have at least a positive (endovascular treatment) or negative (new acute ischemia) effect on POE, respectively. Supplementary Table S2 shows that patients who received endovascular therapy initially were most likely (36%) to receive endovascular therapy again and thus could be treated purely with endovascular therapy; 18% of patients required conversion to a purely open procedure. The question remains to what extent repeated endovascular means impede the chance for distal bypass surgery before eventual major amputation and where the possible turning point in re-occlusion of previous revascularized legs might be. Open surgical treatment by crural bypass is the last option to achieve revascularization. Frequently, these patients have already undergone multiple endovascular pretreatments, so distal connecting vessels decrease significantly in quality with increasing number of procedures, limiting the rate of patency of crural bypasses. Our data show that there are more pretreated, including endovascular, patients over the years. This fact coud be a plausible explanation for 21% amputation rate after open surgical therapy vs. 7% after endovascular therapy in patients with ALI. Hence, the role of specific perioperative medication (including anti-coagulative drugs [29,30]) after endovascular or bypass surgery for ALI needs to be defined more clearly in further studies. Due to the variety of possible combinations of antiplatelet agents (monotherapy, dual therapy, different drugs) and anticoagulation (oral anticoagulation by NOAK, Marcumar or heparin subcutaneously or by continuous infusion), it is extremely difficult to create a meaningful and clear grouping. In addition, comorbidities and resulting indications for platelet function inhibition as well as anticoagulation (especially cardiolgic) must be included in decision making. Until now, therefore, there has been no unanimous opinion in this regard with respect to anticoagulant medication in patients with vascular disease, but over time, a guideline was developed by the ESVS that provides a recommendation in this regard. However, further studies are mandatory [31].

Additionally, we were able to identify preoperative clinical and morphologic variables that were associated with better or worse outcomes. Despite an increase in symptom duration over time and more frequent PAD as pre-diagnosis, there was a significant decrease in the severity of ALI (according to the TASC criteria of ALI, Table 2 and Table S1).

Older age (30 d: aHR 1.17; 180 d: 1.27) and an extended grade of ALI (30 d: TASC IIa aHR 3.29, TASC IIb aHR 3.93, TASC III aHR 7.79; 180 d: TASC IIa aHR 1.97, TASC IIb aHR 2.43, TASC III aHR 4.2), were associated with significantly worse outcomes in the multivariate analysis (Figure 3 and Figure 4). In contrary to our results, Andraska et al. [32] identified female sex to be associated with worse outcomes after ALI regardless of treatment modality in a cohort of 232 patients.

On the other hand, statin therapy (due to hyperlipidemia) was associated with a reduction in the 30-day, 180-day and 5-year mortality and amputation rate (30 d: aHR 0.59, 180 d: aHR 0.65, 5 y aHR 0.65) (Figure 3, Figure 4, and Figure S1). This could be due to the reduced overall mortality in patients receiving statin therapy according to the ESC/EAS guidelines [33]. A large number of meta-analyses have been performed to analyze the effects of statins in different populations (patients from different countries around the world) and in subgroups (e.g., patients with coronary artery disease, patients with diabetes, hemodialysis patients, patients with heart failure) [34]. In the Cholesterol Treatment Trialists (CTT) meta-analysis of individual participant data (IPD) from >170,000 participants in 26 RCTs of a statin vs. control or a more vs. less intensive statin regimen, 34 for each 1 mmol/L reduction in LDL-C, statin/more statin reduced major vascular events (MI, CAD death, or any stroke or coronary revascularization) by ~22%, major coronary events by 23%, CAD death by 20%, total stroke by 17% and total mortality by 10% over 5 years [34].

In addition to the selection of the appropriate surgical or endovascular treatment, clinical and morphologic variables should be included to identify patients at particular risk for increased mortality and amputation. To identify these patients at significantly increased risk, accurate history taking, classification of ischemia and risk stratification are necessary preoperatively.

## 5. Strengths and Limitations

This study on 985 ALI patients over a 17-year period represents one of the largest mono-center studies of its kind. However, several limitations must be mentioned. There is a risk of incompleteness in retrospective data analysis based on electronic patient records harboring possible selection bias. All patients with a principal diagnosis of limb ischemia were closely examined by two independent physicians on the basis of the patient record to exclude miscoding. Patients with lower extremity ischemia were included. There is information on the last follow-up date for all of these patients, so we could confidently say whether the POE was reached or not and could safely perform the analyses. In addition, a sensitivity analysis was performed. Additionally, due to the long time-span of our analyzed data, we were not able to create a more accurate follow-up through contacts with primary care physicians and relatives. This is due to the fact that the data analysis started with patients who were already treated in 2004. Therefore, a sensitivity analysis was performed. The patient cohort and the revascularization procedures are very heterogeneous and were subject to availability and surgeons’ preference. However, we aimed to evaluate the role of changing paradigms (“endo first”) and provide insights into a real-world all-comer cohort over the large time span. By providing a detailed uni-/multivariable analysis on the subject, we have tried to minimize exactly this bias before drawing any conclusions.

Also to be mentioned is that all results are associations and should not be considered causal. To limit the selection bias of a retrospective analysis, we used a multivariable cox model on a large cohort in our analysis and included all patients with 30-day follow up. However, the relationship between endovascular therapy and decreased mortality and/or amputation rates is just an association rather than causal. Since this study is retrospective by nature, future prospective trials including different treatment modalities are mandatory.

## 6. Conclusions

This study of 985 ALI patients confirms the emergent character of acute limb ischemia with a death and/or major amputation rate of 15% after 30 days and 27% after 180 days. Our analysis demonstrates that the treatment approach has changed over time from 2004 to 2020 in favor of endovascular procedures. Statistical analysis suggests that endovascular therapy of ALI might be safer and more effective after 6 months. However, due to the limitations of any retrospective study, further prospective studies are needed to define the safest and most efficient treatment strategy for ALI.

## Figures and Tables

**Table 1:** Patient characteristics.

**Demographics**	n = 985	%
Age (years, median, Q1-Q3)	71 (62–80)	-
Male	555	56.3%
**Symptom onset and biomarker**		
Symptom onset (h, median, Q1-Q3)	24 (6–72)	-
CK on admission (U/L, median, Q1-Q3)	130 (73–412.5)	-
**Severity of acute limb ischemia at admission (TASC criteria)**		
TASC I	265	27%
TASC IIa	347	35%
TASC IIb	331	34%
TASC III	42	4%
**Etiology**		
Arterial thrombosis	629	64%
Arterial thromboembolism	304	31%
Ischemia due to popliteal aneurysm	52	5%
**Occlusion Localization**		
Aorta-iliac	232	24%
Femoral and popliteal arteries	479	49%
Infrapopliteal arteries	274	28%
**Comorbidities**		
Hypertension	687	70%
Smoking	454	46%
PAD	437	44%
CAD	370	38%
Atrial fibrillation	287	29%
Hypercholesterolemia	456	46%
Diabetes	229	23%
COPD	98	10%
Renal insufficiency	179	18%
Previous Stroke	140	14%
Previous Cancer	189	19%
**Previous revascularization (same leg) **	506	51%
**ASA Classification**		
ASA I/II	151	15%
ASA III	388	39%
ASA IV/V	446	45%

ASA, American Society of Anesthesiologists; CAD, coronary artery disease; CK, Creatine kinase (standard value < 170 U/mL); COPD, chronic obstructive pulmonary disease; PAD, peripheral arterial disease; TASC, Trans-Atlantic Inter-Society Consensus, h = hours.

**Table 2:** Temporal trends of ALI characteristics, procedural details and outcomes *.

	Total	1st 200	2nd 200	3rd 200	4th 200	Last 185	*p*	Trend
	985	200	200	200	200	185		
**Severity of acute limb ischemia at admission (TASC criteria)**								
TASC I n (%)	265 (26.9)	49 (24.5)	44 (22.0)	45 (22.5)	59 (29.5)	68 (36.8)	**0.002**	↑
TASC IIa n (%)	347 (35.2)	87 (43.5)	70 (35.0)	84 (42.0)	49 (24.5)	57 (30.8)	**0.001**	↓
TASC IIb n (%)	331 (33.6)	56 (28.0)	80 (40.0)	62 (31.0)	77 (38.5)	56 (30.3)	0.734	↔
TASC III n (%)	42 (4.26)	8 (4.00)	6 (3.00)	9 (4.50)	15 (7.50)	4 (2.16)	0.798	↑
**Etiology**								
Arterial thrombosis n (%)	629 (63.9)	94 (47.0)	103 (51.5)	127 (63.5)	155 (77.5)	150 (81.1)	**<0.001**	↑
Arterial thromboembolism n (%)	304 (30.9)	90 (45.0)	85 (42.5)	67 (33.5)	37 (18.5)	25 (13.5)	**<0.001**	↓
Ischemia due to popliteal aneurysm n (%)	52 (5.28)	16 (8.00)	12 (6.00)	6 (3.00)	8 (4.00)	10 (5.41)	0.143	↓
**Occlusion Localization**								
Aorta-iliac n (%)	232 (23.6)	40 (20.0)	58 (29.0)	54 (27.0)	41 (20.5)	39 (21.1)	0.523	↓
Femoral and popliteal arteries n (%)	479 (48.6)	119 (59.5)	97 (48.5)	98 (49.0)	70 (35.0)	95 (51.4)	**0.006**	↓
Infrapopliteal arteries n (%)	274 (27.8)	41 (20.5)	45 (22.5)	48 (24.0)	89 (44.5)	51 (27.6)	**<0.001**	↑
**Previous revascularization (same leg, any level)**	506 (51.4)	64 (32.0)	86 (43.0)	96 (48.0)	129 (64.5)	131 (70.8)	**<0.001**	↑
**Treatment**								
Open procedures n (%)	535 (54.3)	133 (66.5)	105 (52.5)	118 (59.0)	111 (55.5)	67 (36.2)	**<0.001**	↓
Endovascular procedures n (%)	192 (19.5)	35 (17.5)	26 (13.0)	31 (15.5)	28 (14.0)	73 (39.5)	**<0.001**	↑
Hybrid procedures n (%)	238 (24.2)	30 (15.0)	66 (33.0)	44 (22.0)	56 (28.0)	42 (22.7)	0.260	↔
Primary amputation n (%)	20 (2.03)	2 (1.00)	3 (1.50)	7 (3.50)	5 (2.50)	3 (1.62)	0.458	↔
Primary outcome events (POE) *								
30-day mortality and amputation rate (safety endpoint) n (%)	141 (15.3)	32 (17.7)	31 (16.1)	31 (16.0)	26 (13.8)	21 (12.5)	0.136	↓
180-day mortality and amputation rate (efficacy endpoint) n (%)	201 (27.4)	44 (26.8)	43 (26.7)	46 (30.2)	37 (25.3)	31 (27.9)	0.946	↔
30-day mortality rate	80 (8.66)	15 (8.29)	22 (11.5)	16 (8.25)	15 (7.94)	12 (7.14)	0.376	↓
30-day amputation rate	79 (8.55)	19 (10.5)	15 (7.81)	18 (9.28)	14 (7.41)	13 (7.74)	0.372	↔
**Leg-related complications**								
Surgical site infection n (%)	184 (18.7)	47 (23.5)	31 (15.5)	42 (21.0)	41 (20.5)	23 (12.4)	0.059	↔
Seroma/Lymphatic fistula n (%)	57 (5.79)	25 (12.5)	4 (2.00)	12 (6.00)	11 (5.50)	5 (2.70)	**0.002**	↓
Re-occlusion n (%)	184 (18.7)	32 (16.0)	32 (16.0)	27 (13.5)	31 (15.5)	62 (33.5)	**<0.001**	↑
Acute limb ischemia n (%)	105 (10.7)	16 (8.00)	11 (5.50)	11 (5.50)	14 (7.00)	53 (28.6))	**<0.001**	↑
Wound bleeding n (%)	36 (3.65)	4 (2.00)	9 (4.50)	8 (4.00)	9 (4.50)	6 (3.24)	0.537	↔
**Systemic complications**								
Respiratory complications n (%)	106 (10.8)	18 (9.00)	21 (10.5)	21 (10.5)	25 (12.5)	21 (11.4)	0.332	↑
Renal complications n (%)	88 (8.93)	18 (9.00)	22 (11.0)	19 (9.50)	16 (8.00)	13 (7.03)	0.289	↓
Cardiovascular complications n (%)	125 (12.7)	30 (15.0)	32 (16.0)	28 (14.0)	16 (8.00)	19 (10.3)	**0.020**	↓
Rhabdomyolysis n (%)	47 (4.77)	15 (7.50)	13 (6.50)	4 (2.00)	6 (3.00)	9 (4.86)	0.061	↓

* Thirty-day follow-up was completed by 924 patients; 180-day follow-up was completed for 734 patients. *p* < 0.05 is considered significant and highlighted in bold. Chi-squared Test for Trend in Proportions was done. TASC, Trans-Atlantic Inter-Society Consensus.

**Table 3:** Treatment modalities and crude outcomes.

	n	%
**Open surgical procedures**	**535**	**54%**
Bypass surgery or interposition graft *	168	17%
Embolectomy/thrombectomy *	353	36%
Thrombendarterectomy	14	1%
Percutaneous endovascular transfemoral recanalization *	**192**	**20%**
Local catheter-guided thrombolysis	102	11%
- 24 h	84	82%
- 48 h	18	18%
Angioplasty/Thrombectomy (Ballon-/Stentangioplasty, Aspiration, Rotarex, etc.)	90	9%
Hybrid procedures *	**238**	**24%**
**Primary amputation**	**20**	**2%**
**Additional procedures**		
Fasciotomy	351	36%
- immediately	291	83%
- delayed	60	17%
Minor amputation	6	1%
**Procedure time (hours, median, IQR)**	150.5	(90–260.75)
**Length of stay (days, median, IQR)**	10 (6–18)	-
**Primary outcome events (POE, including single components)**		
30-day-mortality	80	9%
30-day-amputation	79	9%
**Combined 30-day mortality and amputation**	**141**	**15%**
- after open surgical procedure	88	62.4%
- after percutaneous endovascular transfemoral recanalization	12	8.5%
- after hybrid procedure	30	21.3%
- after primary amputation	11	7.8%
**Combined 180-day mortality and amputation**	**201**	**27%**
- after open surgical procedure	122	60.7%
- after percutaneous endovascular transfemoral recanalization	20	10%
- after hybrid procedure	46	22.9%
- after primary amputation	13	6.4%
**Systemic complications (during hospital stay)**		
Respiratory complications	106	11%
Renal complications	88	9%
Cardiovascular complications	125	13%
MACE	33	13.5%
Rhabdomyolysis	47	5%
**Local complications (during hospital stay)**		
Re-occlusion	184	19%
Acute limb ischemia	105	11%
MALE	305	30.5%
Surgical site infection	184	19%
Seroma/lymphatic fistula	66	6%
Wound bleeding	36	4%

* Nine patients received open surgery after unsuccessful endovascular therapy; Hybrid procedures: combined endovascular and open surgical procedures. Thirty-day follow-up was completed by 924 patients; 180-day follow-up was completed for 734 patients.

## Data Availability

The complete, fully anonymized dataset of the “Acute Limb Ischemia” database, owned by the Department of Vascular and Endovascular Surgery, Klinikum rechts der Isar, can be requested from Department for Vascular and Endovascular Surgery and Munich Aortic Center (MAC), University Hospital rechts der Isar, Technical University Munich (TUM), Ismaninger Str. 22, 81675 Munich, Germany.

## Supplementary Materials

The following supporting information can be downloaded at: https://www.mdpi.com/article/10.3390/jcm12175462/s1, Figure S1: Association of ALI localization, treatment modality and complications on combined 5 years’ mortality and major amputation rate (multivariate regression analysis); Figure S2: Kaplan-Meier Curve of combined 5-year mortality and major amputation rate stratified by treat modality; Figure S3: Kaplan-Meier Curve of combined mortality and amputation rate stratified by revascularization modality; Table S1: Temporal trends of patient characteristics; Table S2: Management of early reocclusion during hospital stay; Table S3: Clinical and procedural variables with combined 30-day mortality and amputation rate (safety endpoint, univariate analysis); Table S4: Clinical and procedural variables associated with combined 180-day mortality and major amputation rate (efficacy endpoint, univariate analysis); Table S5: Log-rank statistic for Kaplan-Meier Curves stratified by treatment modality mentioned in Figure 5 and Supplement Figure S2.
