# Supplementary material for: Patients with Acute Limb Ischemia Might Benefit from Endovascular Therapy—A 17-Year Retrospective Single-Center Series of 985 Patients
Source: J Clin Med. 2023 Aug 23;12(17):5462. doi: 10.3390/jcm12175462 (PMC10487798; doi:10.3390/jcm12175462)
Supplement: Supplementary file 1 [file jcm-12-05462-s001.zip › jcm-2535037-supplementary.pdf]

Supplement Table S1: Temporal trends of patient characteristics*.

	total	1st200	2nd 200	3rd 200	4th 200	Last 185	p	trend
	985	200	200	200	200	185		
**Demographics and biomarker**								
Age (years, median, Q1-Q3)	71 (62 – 80)	71 (63 – 81)	69 (61 – 78)	74 (65 – 83)	70 (61 – 80)	71 (63 – 79)	0.417	↔
Male n (%)	555 (56.3%)	113 (56.5%)	115 (57.5%)	105 (52.5%)	119 (59.5%)	103 (55.7%)	0.967	↔
Symptom onset (h, median, Q1-Q3)	24 (6 – 72)	12 (6 – 72)	12 (4 – 72)	20 (6 – 48)	24 (6 – 48)	48 (12 – 140)	**<0.001**	↑
CK on admission (U/ml, median, Q1-Q3)	130 (73 – 412)	136 (65 – 484)	165 (86 – 632)	100 (67 - 349)	130 (79 – 438)	117 (71 – 269)	0.059	↔
**Comorbidities**								
Hypertension n (%)	687 (69.7%)	142 (71.0%)	145 (72.5%)	125 (62.5%)	139 (69.5%)	136 (73.5%)	0.885	↔
Smoking n (%)	454 (46.1%)	65 (32.5%)	99 (49.5%)	87 (43.5%)	105 (52.5%)	98 (53.0%)	**<0.001**	↑
PAD n (%)	437 (44.4%)	53 (26.5%)	73 (36.5%)	84 (42.0%)	110 (55.0%)	117 (63.2)	**<0.001**	↑
CAD n (%)	370 (37.6%)	75 (37.5%)	78 (39.0%)	79 (39.5%)	81 (40.5%)	57 (30.8)	0.308	↔
Atrial fibrillation n (%)	287 (29.1%)	82 (41.0%)	54 (27.0%)	66 (33.0%)	46 (23.0%)	39 (21.1%)	**<0.001**	↓
Hypercholesterolemia and/or statin therapy n (%)	456 (46.3%)	70 (35.0%)	102 (51.0%)	68 (34.0%)	99 (49.5%)	117 (63.2%)	**<0.001**	↑
Diabetes n (%)	229 (23.2%)	45 (22.5%)	43 (21.5%)	43 (21.5%)	54 (27.0%)	44 (23.8%)	0.391	↔
COPD n (%)	98 (9.95%)	22 (11.0%)	27 (13.5%)	16 (8.00%)	19 (9.50%)	14 (7.57%)	0.111	↔
Renal insufficiency n (%)	179 (18.2%)	39 (19.5%)	29 (14.5%)	32 (16.0%)	37 (18.5%)	42 (22.7%)	0.252	↑
previous stroke n (%)	140 (14.2%)	30 (15.0%)	33 (16.5%)	27 (13.5%)	26 (13.0%)	24 (13.0%)	0.338	↓
previous cancer n (%)	189 (19.2%)	32 (16.0%)	51 (25.5%)	43 (21.5%)	35 (17.5%)	28 (15.1%)	0.293	↔
**ASA Classification**								
ASA I/II n (%)	151 (15.3%)	21 (10.5%)	36 (18.0%)	49 (24.5%)	36 (18.0%)	9 (4.86%)	0.221	↔
ASA III n (%)	388 (39.4%)	77 (38.5%)	74 (37.0%)	96 (48.0%)	85 (42.5%)	56 (30.3%)	0.373	↔
ASA IV/V n (%)	446 (45.3%)	102 (51.0%)	90 (45.0%)	55 (27.5%)	79 (39.5%)	120 (64.9%)	0.078	↔
**Outcome**								
Length of hospital stay (days, median, Q1-Q3)	10 (6 – 18)	11 (7 – 19)	10 (6 – 19)	10 (7 – 17)	10 (7 – 17)	10 (6 – 18)	**0.020**	↓

*p<0.05 is considered significant and highlighted in bold. Chi-squared Test for Trend in Proportions was done for categorical variable and Pearson's product-moment correlation was used for continuous variable. ASA, American Society of Anesthesiologists; CAD, coronary artery disease; CK, Creatine kinase (standard value <170U/ml); COPD, chronic obstructive pulmonary disease; PAD, peripheral arterial disease; TASC, Trans-Atlantic Inter-Society Consensus, h=hours.

Supplement Table S2: Management of early re-occlusion during hospital stay.

	n	%
**Patients with early re-occlusion during hospital stay**	**219**	**22%**
No therapy	10	4%
Bypass/Interposition	28	13%
Open thromboembolectomy	37	17%
Thrombendarterectomy	2	1%
Catheter-guided thrombolysis	13	6%
Endovascular thrombectomy	33	15%
Hybrid procedure	64	29%
Major Amputation	32	15%
**Comparison of primary and post-re-occlusion therapy methods**		
**Primary endovascular treatment**	**66**	**34%**
- Change to open procedure	18	18%
- Change to hybrid procedure	17	26%
- Endovascular procedure again	24	36%
- Major amputation necessary	5	7%
- Change to conservative treatment	2	3%
**Primary open surgery**	**95**	**18%**
- Open procedure again	37	39%
- Change to hybrid procedure	24	25%
- Change to endovascular procedure	10	11%
- Major amputation necessary	20	21%
- Change to conservative treatment	4	4%
**Primary hybrid procedure**	**58**	**24%**
- Change to open procedure	12	21%
- Hybrid procedure again	23	40%
- Change to endovascular procedure	12	21%
- Major amputation necessary	7	12%
- Change to conservative treatment	4	6%

Supplement Table S3: Clinical and procedural variables associated with combined 30-day mortality and major amputation rate (safety endpoint, univariate analysis, # of events 141).

	Univariable analysis	
**Demographics and biomarker**	**HR (Cl 95%)**	**p-value**
Age>71 years vs <=71 years	1.44 (1.03–2.02)	**0.031**
Male versus female	0.55 (0.39–0.77)	**<0.001**
Symptom onset >24 h vs <=24 h	0.94 (0.65–1.36)	0.740
CK at admission >130 vs <=130 (U/l)	2.94 (1.97–4.39)	**<0.001**
**Severity of acute limb ischemia at admission (TASC criteria)**		
TASC I	-	-
TASC IIa	4.25 (1.99–9.07)	**<0.001**
TASC IIb	7.79 (3.75–16.2)	**<0.001**
TASC III	25.6 (11.4–57.5)	**<0.001**
**Etiology**		
Arterial thromboembolism	-	-
Arterial thrombosis	0.69 (0.49–0.98)	**0.037**
**Localization**		
Aorta-iliac	1.26 (0.87–1.84)	0.226
Femoral and popliteal artery (Ref.)	-	-
Infrapopliteal arteries	0.57 (0.36–0.89)	**0.014**
**Comorbidities**		
Hypertension	0.78 (0.55–1.10)	0.150
Smoking	0.56 (0.40–0.80)	**0.001**
PAD	0.71 (0.50–0.99)	**0.046**
CAD	1.07 (0.77–1.51)	0.680
Atrial fibrillation	1.40 (0.99–1.98)	0.056
Hyperlipidemia (Therapy with Statins)	0.42 (0.29–0.61)	**<0.001**
Diabetes	1.10 (0.75–1.62)	0.614
COPD	0.99 (0.57–1.72)	0.964
Renal insufficiency	1.49 (1.02–2.20)	**0.042**
Stroke	1.32 (0.86–2.03)	0.210
Cancer	0.70 (0.44–1.11)	0.133
Previous revascularization (same leg, any level)	0.64 (0.46–0.89)	**0.008**
**ASA Classification**		
ASA I/II (Ref.)	-	-
ASA III	0.76 (0.42–1.36)	0.353
ASA IV/V	1.89 (1.13–3.18)	**0.016**
**Treatment**		
Open procedures (Ref.)	-	-
Percutaneus procedures	0.36 (0.20–0.66)	**0.001**
Hybrid procedures	0.74 (0.49–1.12)	0.160
Primary amputation	4.75 (2.53–8.89)	**<0.001**
**Systemic complications**		
Respiratory complications	4.35 (3.03–6.25)	**<0.001**
Renal complications	4.64 (3.19–6.76)	**<0.001**
Cardiovascular complications	5.11 (3.62–7.22)	**<0.001**

Rhabdomyolysis	6.62 (4.32–10.1)	**<0.001**
**Leg-related complications (30 days)**		
Surgical site infection	1.32 (0.90-1.95)	**0.155**
Seroma/Lymphatic fistula	0.11 (0.01-0.76)	**0.026**
Re-occlusion	2.87 (2.05-4.03)	**<0.001**
Acute limb ischemia	3.63 (2.51-5.25)	**<0.001**
Major bleeding	0.35 (0.09-1.41)	0.140

*p<0.05 is considered significant and highlighted in bold. Univariable Cox proportional hazard model was done using Wald Test. ASA, American Society of Anesthesiologists; CAD, coronary artery disease; CK, Creatine kinase (standard value <170U/ml); COPD, chronic obstructive pulmonary disease; PAD, peripheral arterial disease; TASC, Trans-Atlantic Inter-Society Consensus, h=hours, complication – acute limb ischemia means new acute ischemia of the index leg.

Supplement Table S4: Clinical and procedural variables associated with combined 180- day mortality and major amputation rate (efficacy endpoint, univariate analysis, #of events 201)*.

	Univariable analysis	
**Demographics and biomarker**	**HR (Cl 95%)**	**p-value**
Age>71 years vs <=71 years	1.53 (1.16–2.03)	**0.003**
Male versus female	0.53 (0.40–0.70)	**<0.001**
Symptom onset >24 h vs <=24 h	0.91 (0.67–1.24)	0.562
CK at admission >130 vs <=130 (U/l)	2.17 (1.59–2.97)	**<0.001**
**Severity of acute limb ischemia at admission (TASC criteria)**		
TASC I	-	-
TASC IIa	2.57 (1.55–4.26)	**<0.001**
TASC IIb	4.39 (2.71–7.11)	**<0.001**
TASC III	13.0 (7.23–23.5)	**<0.001**
**Etiology**		
Arterial thromboembolism	-	-
Arterial thrombosis	0.67 (0.51–0.90)	**0.007**
**Localization**		
Aorta-iliac	1.33 (0.97–1.85)	0.080
Femoral and popliteal artery (Ref.)	-	-
Infrapopliteal arteries	0.76 (0.54–1.08)	0.131
**Comorbidities**		
Hypertension	0.90 (0.67–1.21)	0.466
Smoking	0.60 (0.45–0.80)	**0.001**
PAD	0.85 (0.64–1.12)	0.245
CAD	1.15 (0.87–1.52)	0.329
Atrial fibrillation	1.39 (1.04–1.86)	**0.026**
Hyperlipidemia (Therapy with Statins)	0.53 (0.40–0.71)	**<0.001**
Diabetes	1.06 (0.77–1.46)	0.723
COPD	1.19 (0.77–1.84)	0.429
Renal insufficiency	1.43 (1.03–1.98)	**0.035**
Stroke	1.30 (0.90–1.88)	0.162
Cancer	0.90 (0.63–1.28)	0.552
Previous revascularization (same leg)	0.80 (0.61–1.06)	0.117
**ASA Classification**		
ASA I/II (Ref.)	-	-
ASA III	0.76 (0.46–1.23)	0.259
ASA IV/V	1.95 (1.26–3.02)	**0.003**
**Treatment**		
Open procedures (Ref.)	-	-
Percutaneus procedures	0.44 (0.27-0.69)	**<0.001**
Hybrid procedures	0.82 (0.58-1.15)	0.239
Primary amputation	4.26 (2.40-7.57)	**<0.001**
**Systemic complications**		
Respiratory complications	4.45 (3.25–6.09)	**<0.001**
Renal complications	4.11 (2.95–5.74)	**<0.001**
Cardiovascular complications	5.37 (4.00–7.21)	**<0.001**

Rhabdomyolysis	5.86 (3.97–8.65)	**<0.001**
**Leg-related complications (30 days)**		
Surgical site infection	1.60 (1.17-2.19)	**0.003**
Seroma/Lymphatic fistula	0.37 (0.15-0.90)	**0.028**
Re-occlusion	2.43 (1.81-3.25)	**<0.001**
Acute limb ischemia	3.09 (2.24-4.28)	**<0.001**
Major bleeding	0.48 (0.18-1.30)	0.149

*p<0.05 is considered significant and highlighted in bold. Univariable Cox proportional hazard model was done using Wald Test. ASA, American Society of Anesthesiologists; CAD, coronary artery disease; CK, Creatine kinase (standard value <170U/ml); COPD, chronic obstructive pulmonary disease; PAD, peripheral arterial disease; TASC, Trans-Atlantic Inter-Society Consensus, h=hours, complication – acute limb ischemia means new acute ischemia of the index leg.

Supplement Table S5: Log-rank statistic for Kaplan-Meier Curves stratified by treatment modality mentioned in Figure 5 and Supplement Figure 2*.

Pairwise comparison using Log-Rank test (30 days)			
	Open procedure	Endovascular procedure	Hybrid procedure
Endovascular procedure	**P < 0.001**	-	-
Hybrid procedure	P = 0.16	**P = 0.035**	-
Amputation	**P < 0.001**	**P < 0.001**	**P < 0.001**

Pairwise comparison using Log-Rank test (180 days)			
	Open procedure	Endovascular procedure	Hybrid procedure
Endovascular procedure	**P < 0.001**	-	-
Hybrid procedure	P = 0.222	**P = 0.011**	-
Amputation	**P < 0.001**	**P < 0.001**	**P < 0.001**

Pairwise comparison using Log-Rank test (5 years)			
	Open procedure	Endovascular procedure	Hybrid procedure
Endovascular procedure	**P = 0.002**	-	-
Hybrid procedure	P = 0.829	**P = 0.006**	-
Amputation	**P < 0.001**	**P < 0.001**	**P < 0.001**

*$p<0.05$ is considered significant and highlighted in bold. Log-rank test was used.

Supplement Figure S1: Association of ALI localization, treatment modality and complications on combined 5 years' mortality and major amputation rate (multivariate regression analysis)*.

5–years mortality and amputation rate	aHR [95% CI]	p-value
**Demographics and biomarker**		
Age per 10 year	1.39 [1.25 - 1.55]	<0.001*
Male	0.77 [0.59 - 1.01]	0.056
Symptom onset	1.37 [1.04 - 1.78]	0.023*
**TASC criteria**		
TASC I	Reference	
TASC IIa	1.76 [1.17 - 2.65]	0.006*
TASC IIb	2.59 [1.72 - 3.91]	<0.001*
TASC III	5.33 [2.95 - 9.64]	<0.001*
**Etiology**		
Arterial thromboembolism	Reference	
Arterial thrombosis	0.77 [0.58 - 1.02]	0.070
Ischemia due to popliteal aneurysm	0.60 [0.28 - 1.27]	0.180
**Comorbidities**		
Hypertension	0.80 [0.60 - 1.07]	0.135
Hyperlipidemia	0.65 [0.49 - 0.88]	0.004*
COPD	1.29 [0.88 - 1.90]	0.191
Previous revascularization	1.55 [1.15 - 2.09]	0.004*
**Treatment**		
Open procedures	Reference	
Percutaneus procedures	0.60 [0.40 - 0.91]	0.017*
Hybrid procedures	1.03 [0.76 - 1.39]	0.867
Amputation	1.23 [0.61 - 2.49]	0.560
**ASA classification**		
ASA I/II	Reference	
ASA III	0.87 [0.55 - 1.37]	0.535
ASA IV/V	1.34 [0.85 - 2.11]	0.203
**Complications**		
Respiratory complications	2.26 [1.61 - 3.16]	<0.001*
Cardiovascular complications	2.45 [1.80 - 3.34]	<0.001*
Seroma/Lymphatic fistula	0.53 [0.29 - 0.98]	0.042*
Reocclusion	1.42 [1.04 - 1.95]	0.028*
Acute limb ischemia	2.15 [1.50 - 3.09]	<0.001*
Woundbleeding	0.44 [0.19 - 1.01]	0.054

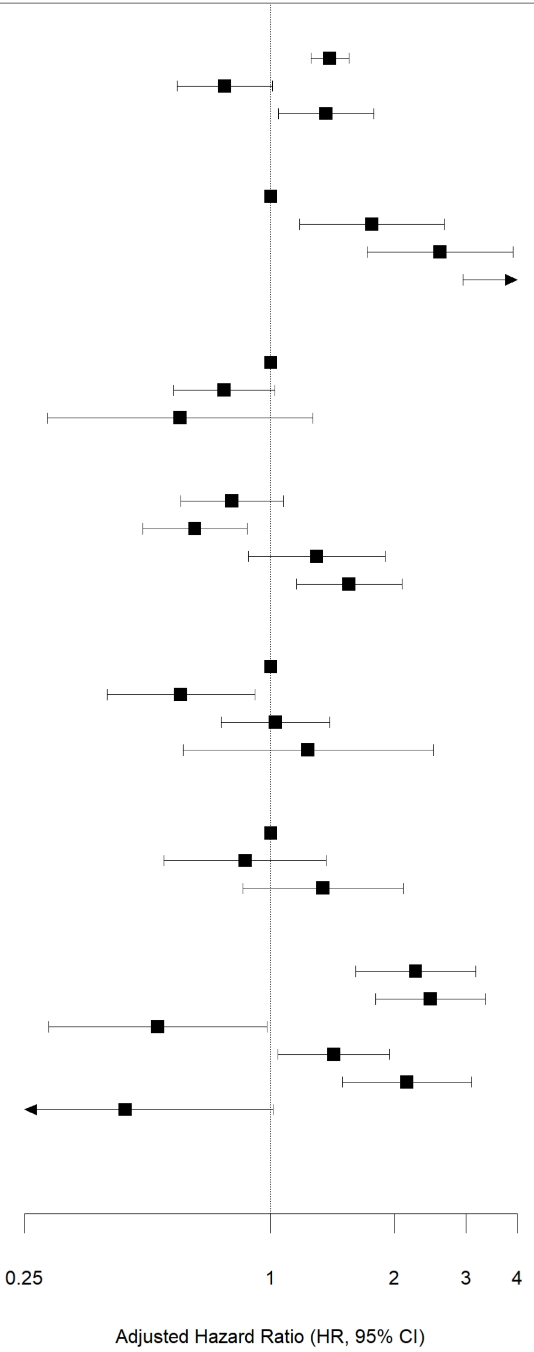

* HR adjusted according to the other variables within the model. No stratification was needed. P<0.05 is considered significant and highlighted with asterisk. Multivariate Cox proportional hazard model was done using Wald Test. ASA, American Society of Anesthesiologists; COPD, chronic obstructive pulmonary disease; PAD, TASC, Trans-Atlantic Inter-Society Consensus, complication – acute limb ischemia means new acute ischemia of the index leg.

Supplement Figure S2: Kaplan-Meier Curve of combined 5-year mortality and major amputation rate stratified by treatment modality*

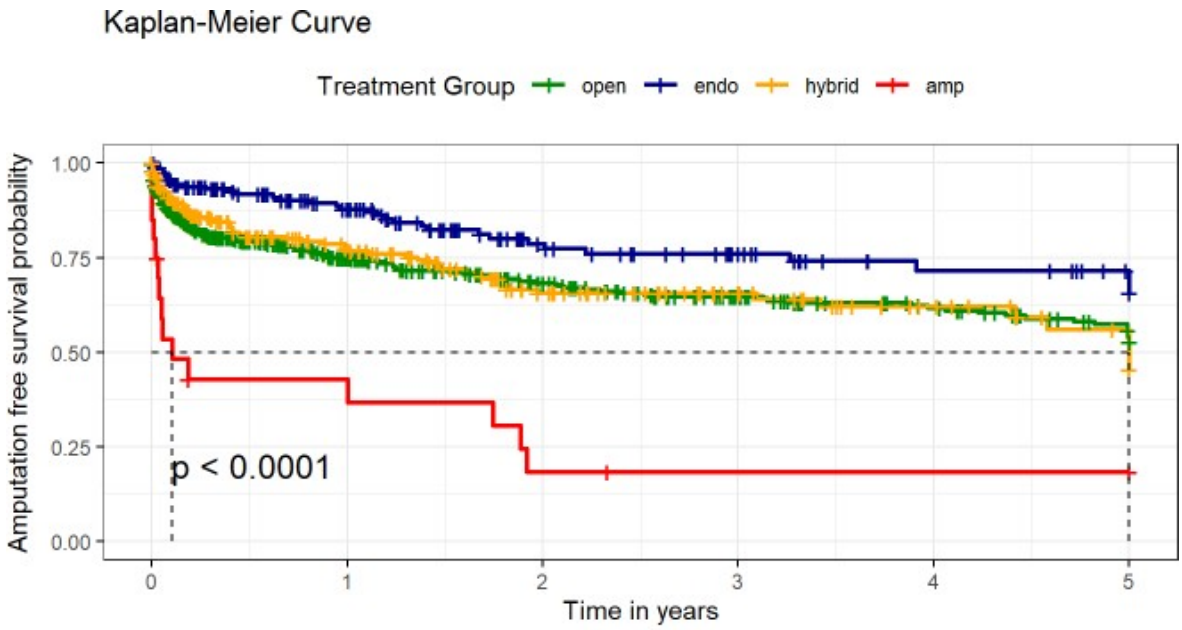

Number at risk

open	535	236	166	120	93	71
endo	192	109	59	43	30	24
hybrid	238	114	64	43	26	16
amp	20	7	3	2	2	2

* p refers to the context of all treatment modalities. $p<0.05$ is considered significant. Log-rank test was used.

Supplement Figure S3: Kaplan-Meier Curves of combined mortality and/or amputation rate stratified by revascularization modality. A) 30-day mortality and major amputation rate (safety endpoint), B) 180-day mortality and major amputation rate (efficacy endpoint) and C) 5-year mortality and major amputation rate*

A

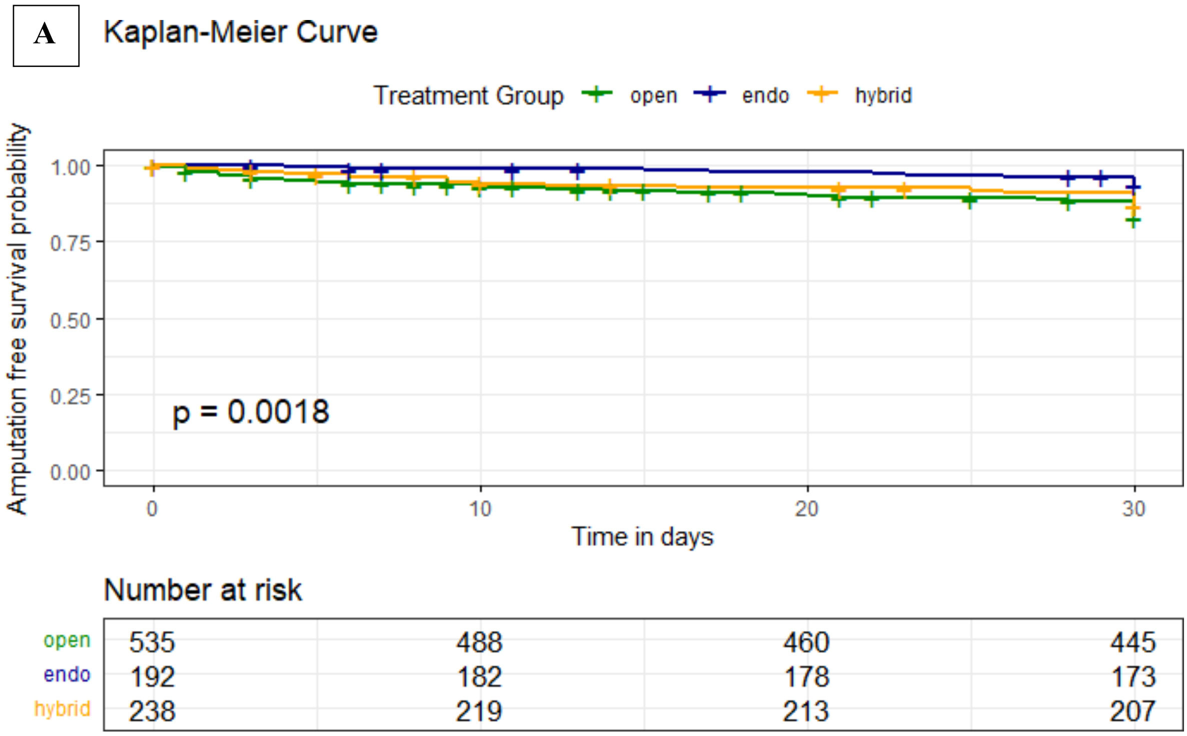

B

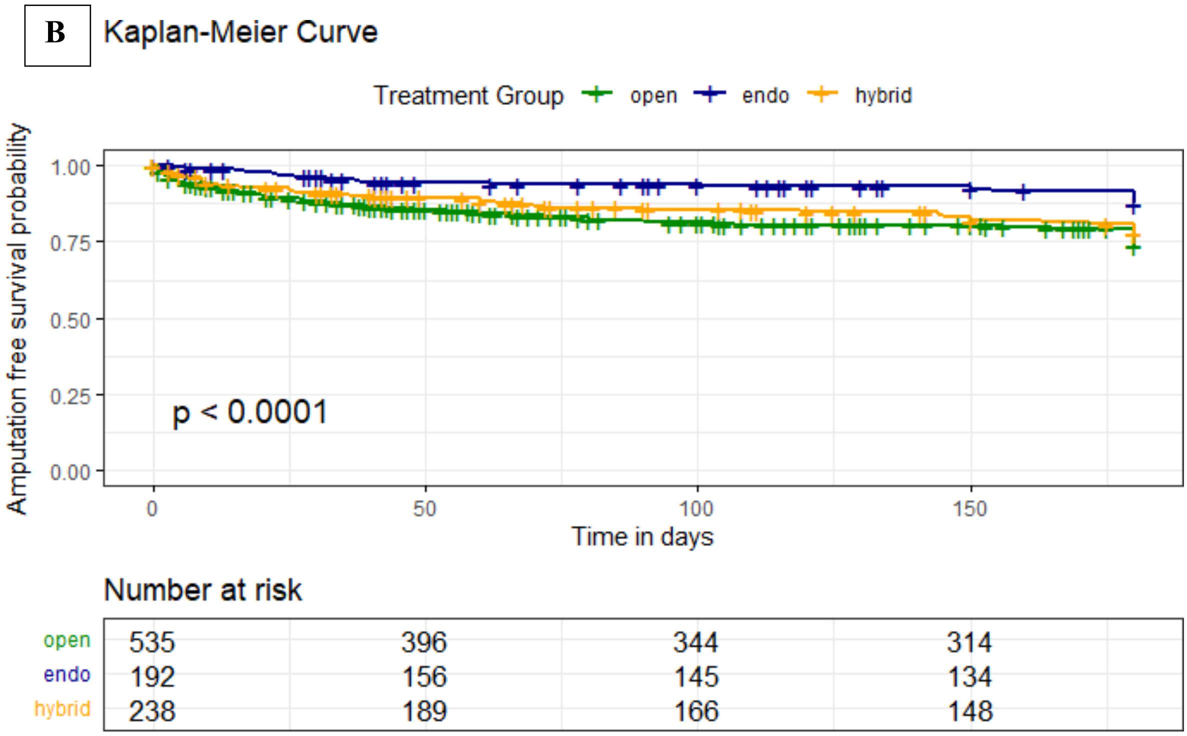

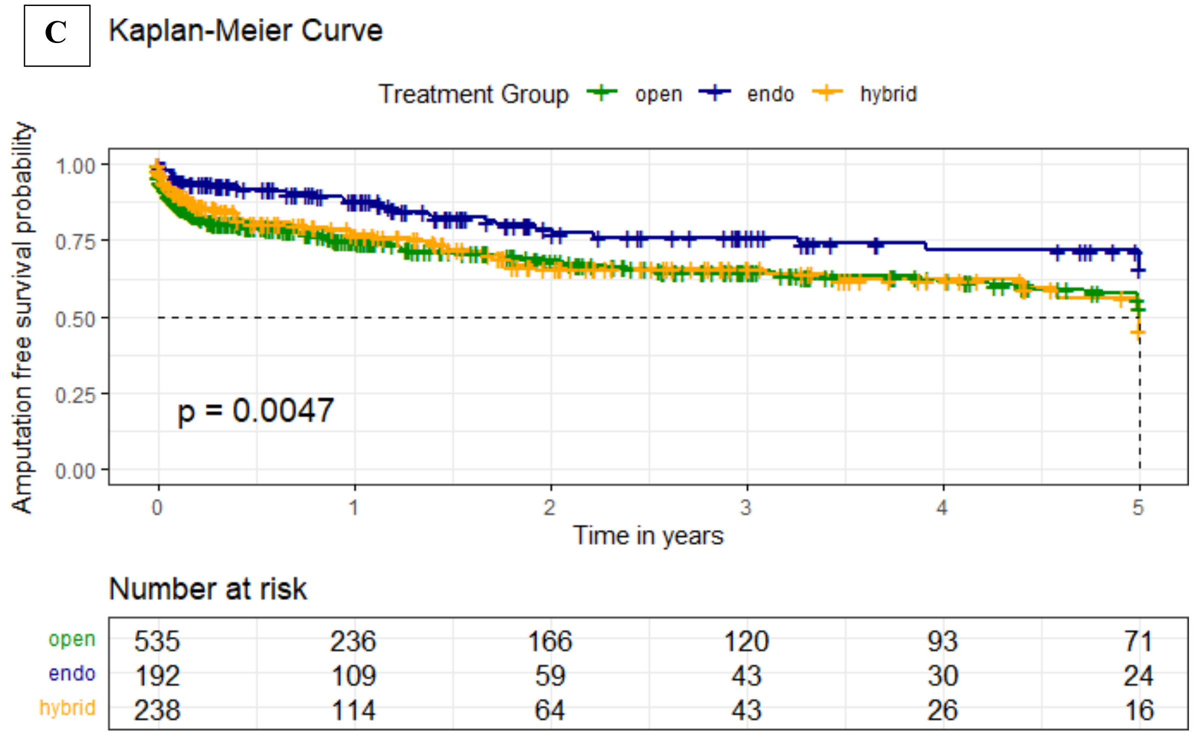

* p refers to the context of all revascularization modalities. p<0.05 is considered significant. Log-rank test was used.
